# Knock-down of ELMO1 in Paediatric Rhabdomyosarcoma Cells by Nanoparticle Mediated siRNA Delivery

**DOI:** 10.5772/62690

**Published:** 2016-01-01

**Authors:** Xinyue Huang, Helen Townley

**Affiliations:** 1 Department of Engineering Sciences, University of Oxford, UK; 2 Department of Obstetrics and Gynaecology, University of Oxford, UK

**Keywords:** Cancer, Metastasis, ELMO1, Nanoparticles, siRNA

## Abstract

Rhabdomyosarcoma (RMS) is the most common soft tissue sarcoma that is found in children and has a poor outcome for those with metastatic disease. Two histological groups have been distinguished - embryonal (ERMS) and alveolar (ARMS) forms. The ARMS subtype has higher rates of metastasis, as well as higher levels of ELMO1, which is thought to be involved in cell migration. Therefore, the knock-down of ELMO1 by targeted siRNA could provide a mechanism to prevent the metastatic behaviour of ARMS cells. However, challenges still lie in the delivery of nucleotides to a tumour site. Herein, we have described the use of a variety of mesoporous silica nanoparticles as a delivery system for siRNA that is specific for ELMO1 and shown the effective reduction in cell invasive behaviour in these cells.

## 1. Introduction

Rhabdomyosarcoma (RMS) is the most common soft tissue sarcoma in childhood and is thought to arise from cells of the skeletal muscle lineage. In patients with localized disease, the overall five-year survival rate has reached approximately 80% due to the combined use of surgery, radiation therapy and chemotherapy [1]. However, for patients with metastatic disease, there have been few improvements, with a five-year event free survival rate of less than 30%.

RMS classically presents as two histological forms -embryonal (ERMS) and alveolar (ARMS). Previous research from our group has shown that the more metastatic ARMS subtype, which has a worse prognosis, also expresses higher levels of the protein ELMO1 [2]. ELMO1 is thought to increase the invasive ability of cells and the downregulation of ELMO1 in ARMS cells has been shown to decrease the invasive nature of the cells. This indicates that ELMO1 may be involved in the migration of ARMS cells and subsequent metastasis. As such, it may be considered as a novel target for the treatment of RMS. The direct regulation of the expression of this protein could be achieved through the use of siRNA molecules. These are small double-stranded RNAs that mediate post-transcriptional gene silencing of a specific target protein by disrupting messenger RNAs containing complementary sequences. However, a major limitation of the use of siRNA molecules both *in vitro* and *in vivo* is the inability of naked siRNA to passively diffuse through cellular membranes.

This is due to the strong anionic charge of the phosphate backbone and consequent electrostatic repulsion from the anionic cell membrane surface [3]. Furthermore, small molecules are vulnerable to being destroyed by the nucleases found in blood or other body fluids. This means that there is a need to pack and protect the siRNA, but with care that this does not affect the biological activity. Despite this, the vast majority of *in vivo* studies using siRNA technology have simply used high doses of nonmodified naked siRNA [4–11]. This means that, to date, most siRNAs in clinical trials are directly administered to local target sites, such as the eye, the skin and the lung (administered by inhalation), thereby avoiding the complexity of systemic delivery. However, most tissues are not accessible and direct injection may be difficult or impossible. Thus, there is a need to develop safer and effective delivery systems. One option for improved delivery is the utilization of nanoparticles as carrier molecules.

Nanoparticles may be prepared from a wide range of organic and inorganic materials. In this study, we have chosen to use mesoporous silica nanoparticles (MSNPs). This is due to the ability to easily control their structure and morphology, and the potential for high surface areas and pore volumes. The silica surface is also densely populated with silanol groups, which can be modified with a wide range of organic functional groups [12], allowing for the modification with moieties such as siRNA. MSNPs have also been shown to degrade over time in aqueous solution [13], resulting in relatively harmless silicic acid. This may be preferable to the use of non-metabolizable nanoparticles, such as carbon nanotubes or gold nanoparticles [14], for long-term use. For the delivery of siRNA to tumours and cells, the external diameter of the nanoparticles is an important factor. Nanoparticles that have been administered systemically have been shown to accumulate in tumours due to the enhanced permeation and retention (EPR) effect [15]. Nanoparticles accumulate in tumours because of the highly permeable blood vessels around tumours. These have large fenestrations (greater than 100nm in size) as a result of rapid defective angiogenesis. Tumours are also characterized by defective lymphatic drainage, which results in the retention of the nanoparticles. At a cellular level, the uptake of nanoparticles is also affected by size, as well as shape and charge. In general, it appears that smaller nanoparticles enter and exit cells more efficiently, as do spherical and positively charged nanoparticles (for review, see [16]).

Therefore, in this study, we have investigated the utility of mesoporous silica nanoparticles (MSNPs) as a delivery system for ELMO1 siRNA in order to prevent the metastatic spread of RMS.

## 2. Materials and Methods

### 2.1 Cell culture

The biological effect of the ELMO1 siRNA was tested *in vitro* on the two paediatric Rhabdomyosarcoma cancer cell lines RD (ATCC code CCL-136) and RH30 (ATCC code CRL-7763), which were obtained from the Marican Type Culture Collection (ATCC; Manassas; VA). The nanoparticles were also tested in Glioblastoma U87-MG cells (ATCC code HTB-14) and breast cancer MCF7 cells (ATCC code HTB-22). The cells were grown in growth medium (Dulbecco's Modified Eagle's Medium (DMEM); Aldrich) supplemented with 10% foetal calf serum (Aldrich), 2 mM L-Glutamine (Aldrich), 100 U/ml Penicillin (Aldrich) and 0.1 mg/ml Streptomycin (Aldrich). They were incubated at 37 °C in a 5% CO_2_ atmosphere. The cells were passaged every three to four days.

### 2.2 Nanoparticle synthesis

Mesoporous silica nanoparticles were prepared according to the method of Hom *et al*. [17] (Hexagonal-symmetry; HMSNP), Nandiyanto *et al*. [18] (Blackberry-like; BMSNP), Zhang *et al*. [19] (Chrysanthemum-like; CMSNP) and Moon *et al*. [20] (Wrinkle-structure; WMSNP). Non-porous silica nanoparticles (SNPs) were prepared according to the method of Rao *et al*. [21]. We have previously fully characterized the particles and these details may be found in Huang *et al*. [13].

### 2.3 Coating of particles with PEI

Polyethylenimine (PEI) was coated onto MSNPs before transfection. In a typical PEI capping experiment, 10 mg MSNPs or SNPs was suspended in 2 mL PEI solution (2.5 mg PEI/mL solution in water, Mw PEI = 24 kD). The suspension was sonicated for 15 min in a water bath and shaken for a further 30 min. The capped nanoparticles were then collected by centrifugation at 8000rpm for 90 seconds and washed with ddH_2_O thrice to remove any uncapped polymer.

To check that the MSNPs were successfully coated with positively charged PEI, the surface charge was determined in physiological buffer (PBS, pH 7.4) before and after coating. The surface charge of the MSNPs was determined to be negative/neutral prior to coating, and positive following the capping procedure. This confirmed the effectiveness of PEI capping.

The surface charge of MSNPs was measured using a Zetasizer Nano ZS (Malvern, UK). To determine the electrokinetic potential, or ζ potential (which represents the surface charge of a colloidal suspension), the uncoated and PEI coated MSNPs were suspended in PBS buffer (pH 7.4) prior to the measurement. Thirty runs were read before the calculation of zeta potential.

### 2.4 Cell viability assay

After the selected cell lines were treated with 0.05 mg/mL PEI coated HMSNPs suspension for 24 hours, the cells were washed twice with pre-warmed PBS. Then, 100 μL 0.5 mg/mL MTT solution (0.5 mg/mL dissolved in a mixture of PBS: growth medium, 1:9 v/v) was added to each well and incubated at 37 °C in darkness. After four hours incubation, the supernatant was removed and the formazan, from the reaction of MTT with viable cells, was solubilized with 100 μL DMSO per well. The absorbance was read at 570 nm using a Tecan INFINITE 200 plate reader. The cell viability was shown to have a linear correlation with absorbance.

### 2.5 siRNA loading efficiency on MSNPs

siRNA was re-suspended in 1x siRNA buffer (five times diluted from 5x siRNA buffer [Dharmacon], comprising 300 mM KCl, 30 mM HEPES-pH 7.5, 1.0 mM MgCl_2_, with RNase-free water), and aliquoted into a 20 μM siRNA stock suspension. Immediately before any further experiment (e.g., transfection), siRNA was loaded on PEI-MSNPs.

In a typical siRNA loading process, a 10 μL siRNA aliquot (20 μM) and 10 μL of 6.25 mg/mL PEI-MSNP suspension was mixed in 5 mL DMEM (no additives) and incubated at RT for 30 min. FITC-conjugated siRNA (Santa Cruz sc-36869) was used to measure the loading efficiency on the siRNA-MSNP delivery system. After FITC-conjugated siRNA loading, the siRNA-MSNP complex was washed once with RNase-free water. Immediately after FITC-conjugated siRNA was loaded, the fluorescence signal of the siRNA-MSNP complex (F_c_), loading supernatant (F_s_) and washed residue (F_r_) was immediately determined after the complex was washed, and every 24 hours up to 72 hours. All of the signals were read using a Tecan INFINITE 200 plate reader. The loading efficiency was calculated by:

$$\frac{F_{c}}{F_{c}+F_{s}+F_{r}}\times 100\%$$

### 2.6 Transfection protocol

The efficacy of the transfection system was initially investigated using a GFP expressing cell line. The U87-MG-cGFP was a kind gift from Dr Karl Morten, NDOG, Oxford. The siRNA transfection procedures were modified slightly from the manufacturer's instructions (Thermo Scientific™ and Santa Cruz Biotechnology™). U87-MG-cGFP cells were plated at a density of 5 × 10^4^ cells/well in antibiotic-free media (DMEM supplemented with glutamine and foetal bovine serum) at 37°C in a 5 % CO_2_ sterile humid incubator and allowed to grow for 24 hours. The cells were either incubated with: (i) buffer alone, (ii) siRNA with no carrier, (iii) PEI-HMSNP, (iv) siRNA with DharmaFECT transfection reagent [Dharmacon™] or (v) siRNA-MSNPs. Immediately before transfection, the siRNA stock solution (10 μM in RNase-free water) was diluted with 1X siRNA buffer (Dharmacon™). For each well, either 2.5 μL siRNA (or the equivalent amount of buffer in controls) was added to 47.5 μL DMEM (serum-free). Separately, 1 μL Dharma-FECT (or the equivalent amount of buffer in controls) was added to 49 μL DMEM (serum-free) in an RNase-free tube. After a five-minute incubation, the two solutions were mixed and incubated for 20 minutes. Subsequently, the cells were washed once with PBS, and 400 μL antibiotic-free media was added to each well before the transfection mixture was added. Therefore, the final concentration of siRNA was 25 nM per well. After a 24-hour transfection period, the cells were washed with PBS twice and GFP fluorescence was measured with a Tecan Infinite 200 plate reader using a well scan mode (7×7 circle; filled) at Ex. 395/Em. 510.

### 2.7 RT-PCR of ELMO1 knock-down

ELMO1 was knocked-down with either ELMO1-targetted siRNA (ON-TARGETplus SMARTpool, human ELMO1, Dharmacon, Thermo Scientific™; sense sequence: 5′-GAAGTTATCAGTCGACATGCCGCCACCCGCGGAC-3′ and antisense sequence: 5′-ATGGTCTAGAAAGCTTTCACATATGAGGGCAGTCC TTTC-3′), transfected with DharmaFECT transfection reagent, ELMO1siRNA-HMSNP or ELMO1siRNA-WMSNP, for 24 hours. A two-step RT-PCR method was used in this study. The total RNA from each sample was purified using ISOLATE II RNA Micro kit (Bioline™) according to the manufacturer's instructions. All of the transfected RH30 or RD cells were washed with warm PBS, trypsinized and collected in an RNase-free tube. Furthermore, 100 μL Lysis Buffer RLY, 2 μL TCEP and 5 μL Carrier RNA working solution (20 ng Carrier RNA) was added to the collected cells separately and the lysate was vortexed robustly twice after each addition. After the lysate was filtered, 100 μL of a 70% ethanol solution was added. The mixture was then placed onto an ISOLATE II RNA Micro Column and centrifuged for 30 sec at 11,000 g. The RNA bonded silica membrane in the column was then desalted with Membrane desalting buffer before the DNase I reaction mixture was added to digest the residue DNA on the membrane. After 15 min DNA digestion at room temperature, the column was washed with Wash Buffer RW1 once and Wash Buffer RW2 twice. Finally, the total RNA was eluted with 30 μL RNase-free water. The total amount of RNA was measured with a NanoDrop 1000 (Thermo Scientific). cDNA was synthesized using a SensiFAST cDNA Synthesis Kit according to the manufacturer's instructions. Briefly, 1 μg purified RNA was mixed with 4 μL 5x TransAmp Buffer, 1 μL reverse transcriptase and RNase free water to make up a 20 μL mixture. Thermal cycling was performed: 25°C for 10 min (primer annealing); 42°C for 15 min (reverse transcription); 85°C for 5 min (inactivation); 4°C hold. The second step of the RT-PCR was then run with the SensiMix II Probe No-ROX kit (BIOLINE™). The testing primer/probe was ELMO1 primer/probe set with a FAM-MGB fluorescence tag (TaqMan; assay ID Hs00404994_m1; Applied Biosystem) and a control ACTB primer/probe set (TaqMan), assay ID Hs01060665_g1, VIC-MGB (Applied Biosystem). According to the supplier instructions, 2 μL template (cDNA synthesized) was mixed with 10 μL 2x SensiMix™ II Probe No-ROX, 1 μL ELMO1 TaqMan primer/probe, 1 μL ACTB TaqMan primer/probe, and 6 μL DNase/RNase-free water.

The samples were then placed in a Rotor-Gene 3000 (QIAGEN) thermal cycler; Cycle 1: 10 min at 95 °C for polymerase activation, Cycle 2 to 46: 10 s at 95 °C then 60 s at 60 °C (every cycle). All of the samples were tested in triplicate.

The results obtained by qRT-PCR were quantified using the comparative threshold method. Giulietti *et al*. [22] detailed this method as ‘the amount of target, normalized to an endogenous housekeeping gene and relative to the calibrator, is then given by 2^−ΔΔCt^, where ΔΔCt = ΔCt_sample_-ΔCt_calibrator_.’ Therefore, in this experiment, the housekeeping gene was β-actin (ACTB), as the ‘calibrator’ in Giulietti's statement; ELMO1 was the ‘sample’ gene. The fold difference of ELMO1 knockout = log (2^−ΔΔCt^), where ΔΔCt = ΔCt_ELMO1_- ΔCt_ACTB_. Here, ΔCt_ELMO1_ equals Ct_ELMO1_ of given sample minus Ct_ELMO1_ of un-knockout sample (blank control) on the same cell line on the same run batch; ΔCt_ACTB_ equals Ct_ACTB_ of given sample minus Ct_ACTB_ of blank control. All of the data were analysed with Rotor-Gene 6000 Series Software 1.7 (QIAGEN) and Microsoft Excel 2010. The fold difference of the ELMO1 expression against ACTB is plotted.

### 2.8 Wound healing assay

In this study, a wound healing assay (scratch test) was performed to evaluate cell migration and invasion. In a typical test, RH30 or RD cells were cultured into a 24-well sterile microplate (Corning). After 24 hours transfection with ELMO1-targetted siRNA on different nanocarriers, the transfected cells were allowed to proliferate, spread and form a confluent monolayer. A 1000 μL pipette tip was used as a pin tool to scratch across the cell layer and remove the scratched content, hence form a cell-free zone in each well.

Images were collected pre- and post- migration. The average width (measured twice across both the vertical and the horizontal scratches) and cell mobility were calculated as:

$$\frac{W_{before}-W_{after}}{W_{before}}\times 100\%$$

## 3. Results and Discussion

**Figure 1.**
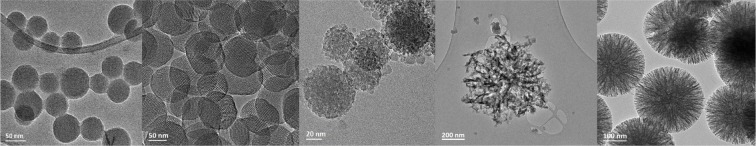
TEM images of MSNPs. Representative images are shown of: (A) Solid nanoparticles (SNPs; 50nm scale bar), (B) Hexagonal mesoporous silica nanoparticles (HMSNP; 50nm scale bar), (C) Blackberry-like mesoporous silica nanoparticles (BMSNP; 20nm scale bar), (D) Chrysanthemum-like silica nanoparticles (CMSNP; 200nm scale bar) and (E) Wrinkle-structured mesoporous silica nanoparticle (WMSNP; 100nm scale bar).

The ability to effectively silence the genes that are involved in metastasis has wide therapeutic potential in the fight against cancer. However, to clinically achieve this, it is necessary for siRNA to be effectively delivered to the tumour site. Unmodified siRNA is unstable in the bloodstream, can be immunogenic and does not readily cross cell membranes [23]. It is vital that the siRNA is delivered to the interior of the target cells in order to be incorporated into the RNAi machinery. However, siRNA molecules are too large and too hydrophilic to cross the cell membrane. Therefore, they require chemical modification or a carrier material to enter the cells. Herein, we explore the use of a silica nanoparticle delivery system. We have previously characterized a number of different MSNPs [13] and their hydrodynamic diameter and surface area are summarized in Table 1. The particles are named in accordance with their physical appearance; Blackberry-like (BMSNP), Chrysanthemum-like (CMSNP), Hexagonal-symmetry (HMSNP), Wrinkle-structure (WMSNP), and solid (SNP; i.e., not porous). The particles increase in diameter in the order: BMSNP< HMSNP<WMSNP< CMSNP. The CMSNPs are most likely too large for systemic administration but may be used for direct intratumoural injection. Meanwhile, the other particles would be an appropriate size to take advantage of the EPR effect. The different morphology particles also show differences in the surface areas such that BMSNP< WMSNP< CMNSP< HMSNP. TEM images of the particles are shown (Figure 1) and it can be seen that the overall shape of all the MSNPs is roughly spherical, which is known to be the preferred shape for cellular uptake. Thus, the shape and exceptionally large surface area of mesoporous particles makes them particularly suitable as carriers for siRNA.

In order to attract the siRNA to the surface of the silica particles, a PEI coating was applied. PEI is a cationic polymer and attracts the negatively charged siRNA so that it is bound by electrostatic interaction to the surface of the particle. The PEI coating on the nanoparticles was too thin to be assessed by TEM (data not shown) but the change in zeta potential (Table 1) clearly indicates the presence of the positively charged polymer on the surface.

**Figure 2.**
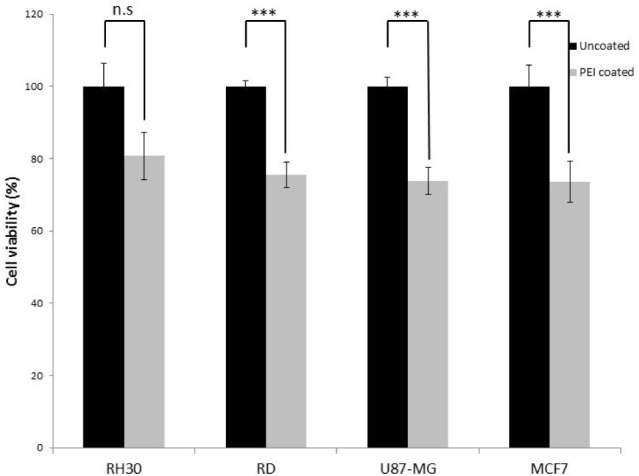
Viability of RH30, RD, U87-MG and MCF7 cells after treatment with PEI coated HMSNP. The cells were treated with the same concentration used in transfection; the viability was assessed with MTT assay. Data are presented as mean± SD of triplicate samples. (n.s. =not significant p>0.02, *p ≤ 0.05, **p ≤ 0.01, ***p ≤ 0.005).

Uncomplexed PEI could potentially be harmful to cells and a number of studies have reported toxicity [24]. Therefore, we assessed the extreme situation, where no nucleotide was bound to the PEI coated nanoparticles. To test this, PEI coated HMSNPs were incubated with a variety of different cell lines. Figure 2 shows that there was a 20% (± 7%) decrease in the cell viability in RH30 cells, although this was statistically insignificant. The cell viability was further decreased in the other cell lines tested by up to 27% (± 6%) in MCF7 cells. However, it is unlikely that all of the sites would be unbound. Therefore, the effects of the PEI would be mitigated (see also Figure 6).

**Table 1.** Summary of MSNP measurements. Hydrodynamic diameter (nm) was assessed using a CPS disc centrifuge and the polydispersity index is indicated. The surface area (m^2^/g) was determined by BET. The zeta potential (mV) of the nanoparticles was assessed before and after coating with PEI (mean ± standard deviation, n=3).

	Hydrodynamic diameter	Surface area	Zeta potential before coating	Zeta potential after coating
HMSNP	98.77 ±1.32	1110.89 ±1.73	−31.43 ±0.61	25.73 ±0.64
BMSNP	57.47 ±1.9	303.02 ±1.00	1.13 ±0.35	18.33 ±0.25
CMSNP	998.81 ±3.2	934.18 ±1.03	−18.00 ±0.62	20.4 ±0.46
WMSNP	234.53 ±1.08	511.56 ±1.99	−59.27 ±2.22	21.55 ±0.23

**Figure 3.**
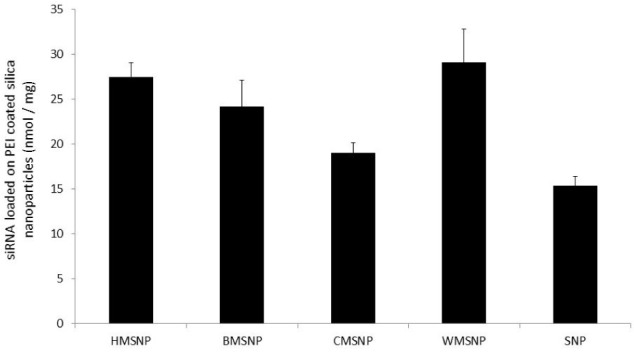
siRNA loading of MSNPS. The different particle morphologies were coated with PEI and incubated with FITC labelled siRNA. The fluorescence signal from the bound siRNA was used to calculate the loading efficiency. Data are presented as mean ± SD of triplicate samples.

To assess the loading of siRNA onto the different MSNPs, FITC-labelled siRNA was incubated with the PEI coated particles. The amount of siRNA bound to each type of particle was then assessed by measuring the fluorescence signal. The data shown in Figure 3 illustrate the variability in the amount of siRNA bound by each of the different morphology MSNPs. These data were used in the subsequent experiment to normalize the amounts of siRNA introduced. Hence, the amount of nanoparticles incubated with cells was varied accordingly.

**Figure 4.**
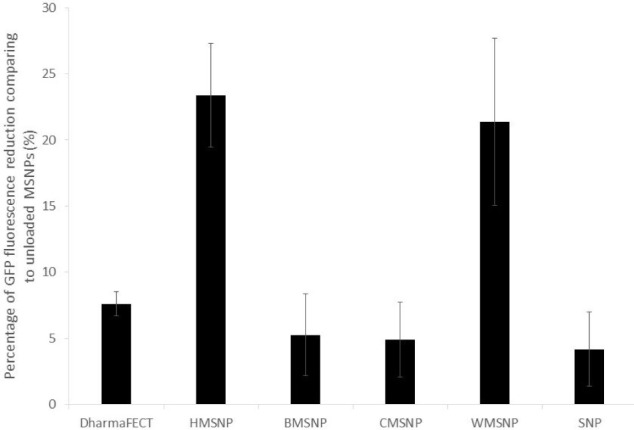
Knock-down of GFP in U87-MG-cGFP cells. The cells were transfected with either DharmaFECT reagent, MSNPs or SNPs. The siRNA was targeted against GFP and a total of 25ng of siRNA was used for transfection. GFP fluorescence was measured and shown as the % reduction compared to the corresponding control. Data are presented as mean ± SD of triplicate samples.

An initial test was devised to assess the ability of the five different MSNPs to deliver siRNA to cells in comparison to DharmaFECT, a standard lipid based transfection agent. The particles were assessed in their ability to introduce GFP-siRNA into GFP expressing U87-MG cells and to reduce the fluorescence signal from the cells. Since the siRNA loading efficiency of the different particle morphologies varied (Figure 3), the number of nanoparticles was normalized so that each cell sample would be incubated with the same amount of siRNA. Of the morphologies tested, BMSNPs, CMSNPs and SNPs were shown to have equal efficacy to DharmaFECT, within error (Figure 4). However, compared to DharmaFECT, the delivery of siRNA with HMSNPs and WMSNPs demonstrated a much greater reduction in GFP fluorescence as a result of the gene knockdown; up to 23 ± 4 % for HMSNPs (p < 0.01), and 21 ± 4 % for WMSNPs (p < 0.01). The low rate of knock-down that is seen with CMSNPs may be due to their size - they are the largest of the particles tested, with a diameter of 754nm (Table 1). The uptake of nanoparticles has been shown to be strongly size-dependent and uptake processes are affected by the bending and stretching forces of the membrane that is involved in endocytosis [25]. BMSNPs have been previously shown to have a positive zeta potential [13]. Since uptake is known to be affected by electrostatic forces [25], this may explain the lower efficacy of these particles in delivering siRNA to the cells. The SNPs are likely to have a much higher ratio of nanoparticles to siRNA. This is because the surface area is much lower due to the lack of porosity. A greater number of nanoparticles may adversely affect the viability of the cell and, subsequently, the siRNA machinery. The HMSNPs and WMSNPs are both a suitable size for cell uptake and have a high surface area for loading of siRNA.

A number of studies have shown hypothesized mechanisms for the cellular uptake of MSNPs, indicating how different nanoparticles may be endocytosed and trafficked in cancer cell lines [25–30]. However, the proposed mechanisms of MSNPs endocytosis would be affected by the cell type, size, surface charge, surface hydrophobicity and shape of MSNPs [28]. A small number of investigations have attempted to track MSNPs in cells. For instance, Sun *et al*. [26] monitored the speed, shape and vertical positions of un-functionalised HMSNPs in live A549 cells using differential interference contrast microscopy. Other investigations on MSNPs endocytosis have been undertaken using TEM (including cryo-TEM) [27–30] and confocal fluorescence microscopy [28, 30]. However, the mechanisms of nanoparticle uptake have not yet been fully elucidated.

Thus, the efficacy of the delivery of the siRNA to the cell machinery is likely to be a function of the efficiency of the uptake of the nanoparticles into the cells. However, this may also vary depending upon the specific cell line.

**Figure 5.**
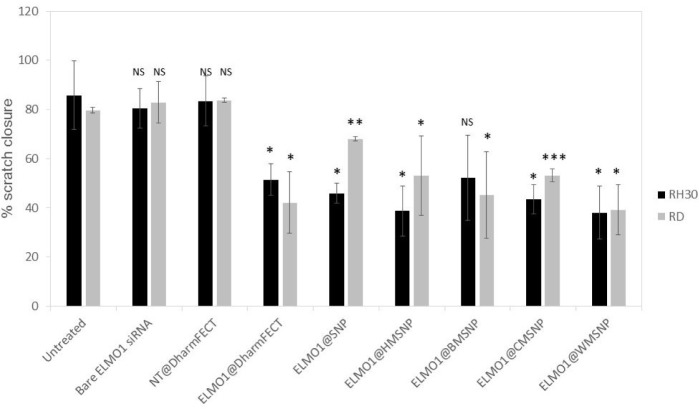
Assessment of invasion after transfection with siRNA. RMS cells were incubated with naked siRNA, non-targeted (NT) siRNA, or ELMO1 targeted siRNA. The lipid transfection agent, DharmaFECT, was compared with MSNPs of different morphologies for efficacy. The percentage closure of a wound inflicted in a confluent cell layer was measured after 24 hours. Data are presented as the mean ± SD of triplicate samples. Significance was tested using a two tailed t-test compared to the untreated cells for each cell line (*p ≤ 0.05, **p ≤ 0.01, ***p ≤ 0.005).

**Figure S1.**
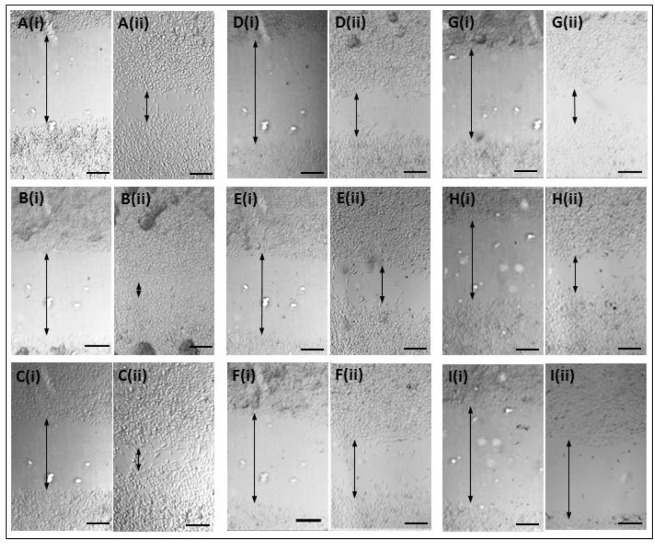
Scratch test showing cell invasion under different test conditions. RH30 cells were transfected with ELMO1 or a non-targeted siRNA for 72 hours. Subsequently, the confluent cell layer was scratched with a pipette tip and the images were recorded at (i) t=0 and (ii) t= 24 hours. Representative images are shown for: (A) Untreated control cells, (B) Bare ELMO1-targeted siRNA, (C) non-targeted siRNA and DharmaFECT, (D) ELMO1 siRNA and DharmaFECT, (E) ELMO1 and SNP, (F) ELMO1 and HMSNP, (G) ELMO1 and BMSNP, (H) ELMO1 and CMSNP and (I) ELMO1 and WMNSP. The arrows indicate cell free gap. Scale bar = 200 μm.

Subsequently, the nanoparticles were applied to our system of interest. The nanoparticles were tested against the paediatric Rhabdomyosarcoma lines, RD (ERMS) and RH30 (ARMS), and coated with siRNA coding for ELMO1. Previous studies have shown that the exogenous expression of ELMO1 in the less metastatic line RD, and in myoblasts which have low/no endogenous ELMO1 expression, increased their invasive nature such that all of the transfected cells were able to migrate [2]. Our studies confirmed that there was a 0.51 fold ± 0.28 (SD) increase in the levels of ELMO1 in RH30 compared to RD (data not shown). Therefore, we first assessed the loaded nanoparticles by use of a wound healing assay. This is a simple method that can be used to mimic the migration of cells *in vivo* [31]. Thus, the assay was used to determine the metastatic potential of RD and RH30 cells after siRNA treatment using the different delivery methods (Figure 5 and S1). The control, untreated RH30 and RD cells showed 85.7% (± 14.0 %) and 79.6% (± 1.2 %) closure of the gap over the duration of the assay, respectively. Bare ELMO1 siRNA was added to the cells and showed no significant difference in gap closure to the controls for either cell type. This may be either due to the breakdown of the unprotected siRNA in the cell culture medium or the inability of the siRNA to penetrate the cell membrane and access the cellular material. As a further control, non-targeting (NT) siRNA was introduced into the cells using the DharmaFECT reagent. This also showed no significant increase in gap closure over the control, with 83.5% (± 10.1 %) and 83.8% (± 1.0 %) gap closure for the RH30 and RD cells, respectively. In the presence of ELMO1 targeted siRNA, in the RH30 cells, all of the MSNP delivery systems were as effective as the DharmaFECT reagent (no significant difference, student t-test shows p> 0.1 in all cases). A similar case was seen for the RD cells (no significant difference, student t-test shows p> 0.2 in all cases), with the exception of the nonporous (SNP) particles. This is a promising outcome, since a reagent, such as DharmaFECT, could not be used for *in vivo* delivery of siRNA. However, an equivalent efficacy illustrates that MSNPs have the potential to be an effective delivery system.

**Figure 6.**
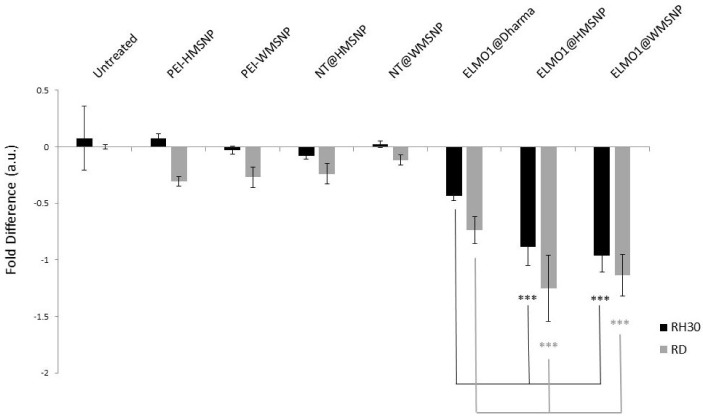
qRT-PCR assessment of ELMO1 knock-down. RMS cells were treated with nanoparticles coated with PEI (PEI- HMSNP/WMSNP), or non-targeting (NT) siRNA, or ELMO1 targeted siRNA. The lipid transfection agent, DharmaFECT, was compared with MSNPs of different morphologies for efficacy. The fold-difference, as determined by qRT-PCR, is shown. Data are presented as the mean ± SD of triplicate samples. Significance was tested using a two tailed t-test compared to the cells treated with ELMO1@DharmaFECT for each cell line (*p ≤ 0.05, **p ≤ 0.01, ***p ≤ 0.005).

To further assess the MSNPs as delivery systems, the gene expression levels were determined. Since HMSNPs and WMSNPs were shown to carry the most siRNA (Figure 3), they were used in the knock-down experiment. The gene expression levels in the control groups, where either no siRNA (cells only, or nanoparticles only) or non-targeting siRNA was administered (NT@MSNP), showed very little fold-change (Figure 6). However, for both the RD and RH30 cell types, there was a statistically significant increase in the gene knock-down when the targeted siRNA was delivered by the nanoparticle carrier (ELMO1@MSNP) compared to lipid transfection with DharmaFECT (p ≤ 0.005). The cells that were incubated with the MSNPs showed approximately double the fold-difference reduction in gene expression that was seen with the lipid transfection reagent. While the knock-down of ELMO1 gene expression after delivery of the targeted siRNA by the MSNPs was almost double that of the DharmaFECT (Figure 6), the MSNP delivery was equally effective as DharmaFECT in the invasion test (Figure 5). This may be due to the fact that the qPCR results do not necessarily correlate with protein levels, and peak levels in repression of mRNA and protein may differ.

There is evidence to suggest that the inhibition of a single step in a metastatic cascade can lead to the suppression of metastasis [21–23]. An integral part of metastasis involves cell migration and this is required at virtually every step of the metastatic cascade. Therefore, ELMO1 may be considered as a candidate for the reduction of metastatic potential, particularly in the ARMS subtype of RMS. Furthermore, MSNPs have been shown to be suitable nanocarriers for siRNA, and that the architecture of the particles affects both the loading with nucleotide and the efficacy of transfection. It would be interesting to further investigate the mechanism of endocytosis for the different nanoparticles, although this is beyond the scope of the present study.

There are a very limited number of siRNA delivery systems that are currently in clinical trials, and none have yet been approved by the FDA. For this to happen, two major bottlenecks must be overcome: the abrogation of off-target silencing effects and efficient delivery of siRNA [32]. We have shown here that nanocarriers, such as MSNPs, have the potential to significantly contribute to the latter.

## 4. Conflict of Interest

The authors declare no conflict of interest.
